# Supplementary material for: Detection of retinal changes with optical coherence tomography angiography in mild cognitive impairment and Alzheimer’s disease patients: A meta-analysis
Source: PLoS One. 2021 Aug 11;16(8):e0255362. doi: 10.1371/journal.pone.0255362 (PMC8357127; doi:10.1371/journal.pone.0255362)
Supplement: S1 File — (DOCX) [file pone.0255362.s002.docx]

| Study | Is the case definition adequate | Representativeness of the case | Selection of controls | Definition of Controls | Comparability of case controls on the basis of the design or analysis | Ascertainment of exposure | Non-Response rate | Quality scores |
| --- | --- | --- | --- | --- | --- | --- | --- | --- |
| Chua 2020 | ☆ | ☆ | ☆ | ☆ | ☆☆ | ☆ | ☆ | 8 |
| Criscuolo 2020 | ☆ | ☆ | ☆ | ☆ | ☆☆ | ☆ |  | 7 |
| Querques 2019 | ☆ | ☆ | ☆ | ☆ | ☆☆ | ☆ | ☆ | 8 |
| Shin 2021 | ☆ | ☆ | ☆ | ☆ | ☆☆ | ☆ | ☆ | 8 |
| Wang 2021 | ☆ | ☆ | ☆ | ☆ | ☆ | ☆ | ☆ | 7 |
| Wu 2020 | ☆ | ☆ | ☆ | ☆ | ☆ | ☆ | ☆ | 7 |
| Yan 2021 | ☆ | ☆ | ☆ |  | ☆☆ | ☆ | ☆ | 7 |
| Yoon 2019 | ☆ | ☆ | ☆ | ☆ | ☆☆ | ☆ | ☆ | 8 |
| Zhang 2019 | ☆ | ☆ | ☆ | ☆ | ☆ | ☆ |  | 6 |

Newcastle-Ottawa Quality Assessment Scale Case Control Studies
